# Newfangled Topical Film-Forming Solution for Facilitated Antifungal Therapy: Design, Development, Characterization, and In Vitro Evaluation

**DOI:** 10.3390/polym15041003

**Published:** 2023-02-17

**Authors:** Bhakti Dhimmar, Rahul Pokale, Mohamed Rahamathulla, Umme Hani, Mohammad Y. Alshahrani, Sultan Alshehri, Faiyaz Shakeel, Prawez Alam, Riyaz Ali M. Osmani, Amit B. Patil

**Affiliations:** 1 Department of Pharmaceutics, JSS College of Pharmacy, JSS Academy of Higher Education and Research (JSS AHER), Mysuru 570 015, Karnataka, India; 2 Department of Pharmaceutics, College of Pharmacy, King Khalid University, Abha 61421, Saudi Arabia; 3 Department of Clinical Laboratory Sciences, College of Applied Medical Sciences, King Khalid University, Guraiger, Abha 61421, Saudi Arabia; 4 Department of Pharmaceutical Sciences, College of Pharmacy, AlMaarefa University, Ad Diriyah 13713, Saudi Arabia; 5 Department of Pharmaceutics, College of Pharmacy, King Saud University, Riyadh 11451, Saudi Arabia; 6 Department of Pharmacognosy, College of Pharmacy, Prince Sattam Bin Abdulaziz University, Al-Kharj 11942, Saudi Arabia

**Keywords:** drug delivery, polymers, fungal infections, luliconazole, topical film forming solution, in vitro antifungal activity, *Candida albicans*

## Abstract

Luliconazole is a broad-spectrum topical antifungal agent that acts by altering the synthesis of fungi cell membranes. Literature suggests that the recurrence of fungal infection can be avoided by altering the pH of the site of infection. Studies have also suggested that fungi thrive by altering skin pH to be slightly acidic, i.e., pH 3–5. The current study is aimed to design, develop, characterize, and evaluate an alkaline pH-based antifungal spray solution for antifungal effects. Luliconazole was used as an antifungal agent and an alkaline spray was formulated for topical application by using Eudragit RS 100, propylene glycol (PG), water, sodium bicarbonate, and ethanol via solubilization method. Herein, sodium bicarbonate was used as an alkalizing agent. Based on DSC, FTIR, PXRD, scanning electron microscopy (SEM), and rheological analysis outcomes, the drug (luliconazole) and polymer were found to be compatible. F-14 formulation containing 22% Eudragit RS 100 (ERS), 1.5% PG, and 0.25% sodium bicarbonate was optimized by adopting the quality by design approach by using design of experiment software. The viscosity, pH, drying time, volume of solution post spraying, and spray angle were, 14.99 ± 0.21 cp, 8 pH, 60 s, 0.25 mL ± 0.05 mL, and 80 ± 2, respectively. In vitro drug diffusion studies and in vitro antifungal trials against *Candida albicans* revealed 98.0 ± 0.2% drug diffusion with a zone of inhibition of 9 ± 0.12 mm. The findings of the optimized luliconazole topical film-forming solution were satisfactory, it was compatible with human skin, and depicted sustained drug release that suggests promising applicability in facilitated topical antifungal treatments.

## 1. Introduction

Fungal infection, commonly known as “mycosis”, is a skin infection caused by a fungus. Fungi, which dwell in the earth, on plants, household surfaces, and skin, can trigger skin problems, such as rashes or bumps. Fungi typically grow in moist parts of the body such as between the toes, in the vaginal area, and beneath the breasts, where they can cause skin irritation, scaling, redness, itching, swelling, or blister formation [1]. Dermatophytes (such as *Epidermophyton*, *Microsporum*, and *Trichophyton*) and yeasts (such as *Candida* or *Malassezia furfur*) cause most common fungal skin diseases. These fungi reside only in the outermost layer of the epidermis (*Stratum corneum*) and do not penetrate into deeper layers [2].

In topical spray, a polymeric solution of the drug is sprayed over the intact skin to get a sustained release of the drug from the polymeric matrix in which the drug is in dissolved form. The drug diffuses slowly, through the polymer matrix as the organic solvent vehicle evaporates and passes through the skin barrier. The type of nozzle, the pressure applied on the spray, the size of the aperture, and the nature of the liquid are major factors that influence the spray ability of film-forming solutions (FFSs). The pH, viscoelastic, in situ and thermal-sensitive properties of FFS are essential to study and to determine various aspects required to be considered while selecting solvents, polymers, and other excipients [3].

A film-forming solution (FFS) is a sprayed drug-delivery system that forms a film when it comes into contact with the target therapeutic region by using the polymer as a matrix for film formation. Following the formation of the film, the drug-release process is similar to that of a patch, in that the polymer matrix carrying the medication will release it in a sustained manner [4]. Unlike topical patches and other topical medicines, however, films form in the pattern of the skin or wound because deep indentations can be exposed to minute droplets of the film-forming solution. Naturally, this considerably enhances drug delivery to the target tissue. When the film-forming system is applied directly to the skin, it evaporates the solvent and forms a thin, transparent film in situ. The composition of the film-producing system changes dramatically after application to the skin due to the loss of the volatile components of the vehicle, resulting in the creation of residual film on the skin surface. The concentration of the drug on the skin surface increases during this phase, reaching saturation and maybe supersaturation. By raising the thermodynamic activity of the formulation without compromising the skin’s barrier, supersaturation results in increased medication flow through the skin, decreasing side effects or irritation. Drug dosages in a film-forming spray can also be varied based on the volume of solution in each spray to control systemic or local effects. An FFS also ensures that medications are distributed evenly and effectively. Patient compliance can also be increased through ease of use. This thin and non-sticky film also improves patient comfort throughout activities as compared to patches, ointments, gels, and other similar products, which have a gritty and sticky texture when applied. The thin film also allows wound moisture to permeate, allowing the equilibrium to be maintained. Inappropriate wound humidity, as with the use of patch treatments, might induce infection or irritation. The film-forming fluid is sprayed by using any type of sprayer to produce droplets. Each sprayer has distinct specs and intended functions, but they all have medicinal applications in mind [5,6,7].

Luliconazole (LCZ) is a new topical antifungal imidazole, approved by the US FDA in November 2013, that has broad-spectrum and effective antifungal activity and is used to treat an array of fungal infections. However, it has water solubility and skin permeability limitations. LCZ exhibits the lowest minimum inhibitory concentration (MIC) among many other tested antifungal drugs, including sertaconazole, clotrimazole, neticonazole, miconazole, bifonazole, ketoconazole, and eberconazole. LCZ acts by inhibiting the fungal lanosterol 14-α demethylase enzyme, which results in ergosterol depletion and accumulation of toxic sterols in fungal cell membrane, consequently leading to fungal cell lysis. Moreover, it also inhibits a few essential enzymes thereby interfering with mitochondrial electron transport processes and energy production. In clinical trials of LCZ, no serious toxicity was reported, and only local irritation (mild contact dermatitis and cellulitis) at the site of application was found. LCZ is an effective and preferable antifungal active pharmaceutical ingredient owing to its potent antifungal activity against a wide range of fungi [8,9].

LCZ has lower aqueous solubility that limits dermal bioavailability and acts as a barrier to topical delivery. The solubility of the drug in the lipid phase of *Stratum corneum* also acts as a rate-limiting step for the permeation. Fungal infection involves the epidermis and dermis, as well as deeper layers of skin that require customizing the drug delivery in such a way that will localize high drug concentrations at the epidermis and dermis layers. However, the commercial topical formulation of LCZ (1%*w*/*v* cream LUZU^®^) is associated with lower skin permeation and shorter skin retention of the drug [10,11,12]. In recent years, topical FFSs have gained great importance as a potential drug carrier for topical delivery due to their unique advantages and great versatility as compared to conventional formulations. FFSs have gained increasing interest and are found to be superior to other topical drug delivery systems owing to their versatile characteristics such as the use of minute quantities of excipients, high drug-payload capacity, lower toxicity, higher chemical stability, and easy scale-up and manufacturing.

The present research aims at designing, formulating, and evaluating an alkaline antifungal in situ FFS spray containing LCZ for providing improved drug penetration and skin retention at the site of infection and for effective management of fungal infections with improved patient compliance. F14 formulation was optimized using the quality by design (QbD) approach implying the design of experiment software V-13, from among all the prepared formulations. The essential parameters like viscosity, pH, drying time, the volume of solution post spraying and spray angle were studied in detail. The effectiveness of optimized formulation was further proven via in vitro drug-diffusion studies utilizing a cellophane membrane, and in vitro antifungal trials against the *Candida albicans* strain.

## 2. Materials and Methods

### 2.1. Materials

Luliconazole (LCZ), sodium bicarbonate, sodium chloride, and sodium hydroxide were procured from Yarrow Chemicals (Mumbai, India). Eudragit^®^ RS 100 was procured from Vikram Thermal (Ahmedabad, India). Propylene glycol (PG) was procured from LOBA Chemie (Mumbai, India). Ethanol and other solvents were procured from Merck (Mumbai, India). The rest of the chemical compounds, reagents, and solvents used were of analytical grade and were used as procured according to the manufacturer’s instructions.

### 2.2. Methods

#### 2.2.1. Preparation of Topical FFS

The polymeric solution was prepared by using LCZ as an antifungal agent, Eudragit RS 100 as a polymer, PG as a plasticizer, sodium bicarbonate as an alkalizing agent, and a solvent such as ethanol by solubilization method. Film-forming solutions were prepared by adding different concentrations of Eudragit RS 100 into ethanol and the solution was stirred overnight for complete swelling of the polymer. PG in varying concentrations was added to the above-prepared polymeric solution. LCZ was added to the prepared formulation and was stirred for another 24 h. Sodium bicarbonate solution was prepared and added to the above solution to make it alkaline [13].

#### 2.2.2. Preparation of Alkaline Polymeric Solution for Topical Spray

To formulate an alkaline formulation different alkalizing agents such as sodium hydroxide and sodium bicarbonate were used along with sodium chloride electrolyte solution. All the alkalizing agents were added into water to get the 0.02% concentration followed by addition into the polymeric solution and determination of the pH of the solutions. The solutions components like sodium chloride and sodium hydroxide have been reported to impart brittleness and increased drying time of the film, and sodium bicarbonate had shown high compatibility; hence used in the preparation of alkaline polymeric solution [14].

#### 2.2.3. Design of Experiment (DoE)

A full factorial design was used to optimize formulated in situ polymeric films. Historical data design in response surface methodology (RSM) with Box–Behnken design was used. A total of 17 runs were generated for polymer, drug, plasticizer, and alkalizing agent combinations. Drug, polymer, alkalizing agent, plasticizer, and solvent were selected as independent variables, whereas, drying time, pH, and drug diffusion were selected as dependent variables [15]. The obtained results were then studied for significant changes when variables were changed within their limits, as shown in Table 1.

### 2.3. Preformulation Studies

#### 2.3.1. Differential Scanning Calorimetry (DSC)

DSC study was conducted to check the thermal stability of luliconazole and the physical mixture of luliconazole with Eudragit RS 100. The physical mixture was analyzed by using a differential scanning calorimeter (DSC-60, Shimadzu, Kyoto, Japan). In this method, the heat energy was introduced into a sample cell and a reference cell simultaneously. The temperature for scanning the sample was set in the range of 20 to 200 °C with a heating level of 10 °C/min in the N_2_ (nitrogen) atmosphere. Empty cells (alpha-alumina discs) of high purity were used as a reference for calorimetric measurements. Approximately 5 mg of the sample was taken for the analysis, and scanning was performed [16,17].

#### 2.3.2. X-ray Diffractometry (XRD)

The XRD analysis of pure luliconazole, polymer, and the physical mixture was performed at room temperature, and the patterns were obtained by using a Rigaku diffractometer. The patterns of diffraction were obtained by Ni-filtered Cu Kα radiation, where λ = 1.498° A, under 20 mA current, and 40 kV voltage operation [18]. The samples were screened across the 10° to 90° 2θ range, with experimental parameters set as a scan step size of 0.02° for 2 s with a scan speed of 0.01°/S.

#### 2.3.3. Fourier-Transform Infrared (FTIR) Spectroscopy

Fourier-transform infrared spectra of LCZ with Eudragit RS 100 were recorded by using the FTIR spectrophotometer (FTIR-8400s, Shimadzu, Kyoto, Japan). This analysis was performed to check the compatibility between the components of the physical mixture, and it also helps in identifying new bond formation. The physical mixture was taken and mixed with potassium bromide (KBr) powder in a ratio of 1:4. The mixture was pressed into pellets and analyzed. The FTIR spectra of pure LCZ and a mixture of LCZ with Eudragit RS 100 were obtained in the range of 400 to 4000 cm^−1^ at room temperature [19,20].

### 2.4. Evaluation of Polymeric Spray Solution

#### 2.4.1. Appearance

The FFS was studied for physical characteristics such as colour, odour, and appearance by visual inspection and testing.

#### 2.4.2. pH

A digital pH meter (SevenExcellence S500, Mettler-Toledo, Columbus, OH, USA) was used to determine the pH of the spray solution. The pH meter was calibrated with phosphate buffer pH 4.0, 7.0, and 10 before evaluating the pH of all the formulations. The pH was then measured by dipping the electrode of the pH meter into 20 mL of spray solution for a minute. The pH of each formulation was measured three times and the mean values were reported [21].

#### 2.4.3. Viscosity

Brookfield viscometer (DV-II, LV model, Brookfield, WI, USA) was used to measure the viscosity by using small volume adaptor with a thermos stated water jacket and ULA-S00 spindle. A total of 20 mL of the sample was taken in a ULA cylinder and the spindle was rotated at 10 rpm speed at 25 ± 1 °C. The samples were equilibrated for 10 min before the measurement; moreover, the instrument was equipped with a temperature control unit. An average of three readings was taken for all the formulations [22].

#### 2.4.4. Rheological Study

The rheological studies were performed on optimized polymeric film and placebo formulation solution by using MCR100 controlled stress rheometer (Anton-Paar Gmbh, Graz, Austria), having a coaxial cylinder with a radii ratio of 1.08477. The rheometer had a temperature-controlled unit for maintaining the temperature with a precision of 0.01 °C and a flowing water jacket was a part of the assembly used. The rheological factor, shear stress (Pa) was assessed straightly, rising to a shear rate of 250 S^−1^ with a 20-S interval and 25 shear stresses. Moreover, shear stress (Pa) was assessed by using US200 (Paar-Physica, Anton-Paar Gmbh, Graz, Austria) software. The rheological measurements at two different temperature levels (25 °C and 37 °C) were carried out corresponding to the storage area and the body temperature, respectively. All experiments were performed in triplicate, with a new sample taken for each assessment [23].

#### 2.4.5. In Vitro Diffusion Study

In vitro diffusion studies for the polymeric spray were carried out by cellophane membrane by using a Franz diffusion cell. The cellophane membrane (Cellulose Tubing, Sigma Diagnostics, St. Louis, MO, USA; molecular weight cutoff, 1000 Da) was activated by soaking it overnight in a phosphate buffer solution (PBS) of pH 6.8. The membrane was kept between the donor and receptor compartments. In the donor compartment, the polymeric solution was transferred, whereas the receptor compartment had phosphate buffer, and the entire surface of the membrane was in contact with PBS. The samples were withdrawn from the receptor compartment at intervals of 0, 1, 2, 3, 4, 5, 10, 15, 30, 60, 120, 180, 240, 300, 360, and 420 min, and the specific amount of sample was collected at each time interval and an equal volume of fresh phosphate buffer was replaced each time. The amount of drug diffused was measured by using a UV spectrophotometer (UV-1800, Shimadzu, Kyoto, Japan) at 290 nm (Supplementary Figures S1 and S2, Table S1) [24].

#### 2.4.6. In Vitro Antifungal Activity

In vitro antifungal activity of prepared alkaline polymeric solution for spray was performed by using the well diffusion method and disc diffusion method. *Candida albicans* were used as a fungal strain. Subculture of *Candida albicans* (ATCC90028) was acquired from June Enterprises Pvt. Ltd., Navi Mumbai, India. Sabouraud dextrose agar (SDA) powder was mixed in distilled water to prepare agar media. Mueller–Hinton broth powder was dissolved in distilled water with agar and glucose powder for the preparation of Mueller–Hinton broth and sterilized by using an autoclave. The prepared media was poured aseptically into the petri dish. In this study, appropriate dilutions (4 × 10^−5^ CFU/mL) of 0.1 mL of *C. albicans* were then mixed into the petri dish at 35 °C and were kept for 48 h. Known concentrations of the samples were loaded into the well and incubated in an inverted position. A total of 1 mL of blank formulation (negative control), optimized formulation ≅ to 10 mg of the drug (test), and marketed product ≅ to 10 mg (positive control) were placed in the respective plates. The entire process of operation was carried out in an aseptic condition. The plates were incubated at 35 ± 1 °C for 24 h. Post 24 h, the zone of inhibition of the test organism, i.e., inhibition of growth around each well was measured in mm and the in vitro antifungal activity of optimized formulation toward *Candida albicans* was reported [25].

#### 2.4.7. Stability Studies

The optimized polymeric spray was stored for one month at 30 ± 3 °C away from light, and evaluation tests were performed for 0, 7, 14, 21, and 28 days. The polymeric spray was subjected to various tests like viscosity, drying time, pH, and in vitro drug permeation studies. The methodology followed for the study was the same as described above [26].

### 2.5. Evaluation of Polymeric Film

#### 2.5.1. Drying Time

The time taken by the polymeric solution to dry on a glass slide or the hand arm is referred to as drying time. The drying time was recorded by using a digital stopwatch [27].

#### 2.5.2. Films Outer Surface Characterization

The outer surface of the dried films was evaluated for its stickiness and smoothness by pressing and rubbing absorbable cotton wool on the dry film under low manual pressure. After pressing and rubbing with cotton, if the fibers from the cotton stick to the outer surface of the film, then it was termed sticky and if no fibers stuck, the outer surface was said to be smooth [28].

#### 2.5.3. Determination of Transparency

Various concentrations at 22, 23.5, and 25% of the polymer, and plasticizer at 1.5, 2.25, and 3% were used to determine the appropriate concentration required to form a transparent, shiny, and continuous film. From these experiments, we could conclude that of 22% concentration of Eudragit RS 100 and PG 1.5% were required to obtain a film with satisfactory characteristics. The 23.5% concentration of Eudragit RS 100, although sprayable, was unable to form a continuous film over the skin and glass slides due to insufficient polymer concentration. In addition, the 25% concentration of Eudragit RS 100 was unable to be sprayed and unable to form a continuous film over the skin and glass slides due to higher polymer concentration.

LCZ could be incorporated in sprays at 22% polymer and 1.5% plasticizer concentration to obtain a satisfactory film. The film was transparent, clear, elegant, and aesthetic in appearance; there was no entrapment of air, the nature of the film was flowable, and the film formed was not highly viscous. Transparent, shiny, non-messy, nongreasy, non-staining, and nonflaky films were obtained by using Eudragit RS 100 22% as a film-forming polymer and PG 1.5% as plasticizer.

The dried films were determined by visual inspection and were graded based on their transparent nature like completely transparent, acceptable, or blurred. The films were also inspected for any bubble entrapment and deformation. Grading was as follows:

++, completely transparent;

+, acceptable; and

−, blurred.

#### 2.5.4. Uniformity of Film Thickness

A hand-held micrometer (Mitutoyo Corporation, Kanagawa, Japan) was used to calculate the film thickness with 0.01 mm precision. The average thickness of film samples was determined at four to six random points, and values were recorded [29].

#### 2.5.5. Folding Endurance

Folding endurance was determined by repeatedly folding the film from the same point before it broke. This is an indication of the film’s brittleness.

#### 2.5.6. Tensile Strength

A universal tensile test machine (International Equipments, Mumbai, India) was used to determine the peak load and tensile strength of the film. The tensile strength was calculated by using the following formula [30]. Results were expressed in megapascals (MPa) and reported to a significant figure. We have

(1) $$Tensile\;strength=\;\;\frac{\left(load\;at\;break\right)}{\left(original\;width\right)\left(original\;thickness\right)}.$$

#### 2.5.7. Scanning Electron Microscopy (SEM)

A scanning electron microscope (JEOL JSM-IT300, Tokyo, Japan) was used to determine the microstructure of the films. Film samples were packed in an airtight desiccator containing silica gel for two weeks. The cross-section of the sample was then evaluated by using films that had been randomly and gently immersed in liquid nitrogen [31,32]. On copper stubs, gold-coated sample films and solutions were mounted and then observed by using a 10-kV step-up voltage.

### 2.6. Container Evaluation

#### Volume of Solution upon Each Actuation

The amount of polymeric spray solution delivered with each actuation was determined by using Equation (2) [33],

(2) $$AL=(W_{t}-W_{0})\;D_{n},$$

where, *AL* is the volume of solution upon each actuation, *W*_0_ is the initial weight of the formulation before actuation, *W*_t_ is the weight of formulation after actuation, and *D*_n_ is the density of the solution.

### 2.7. Statistical Analysis

All the recorded research data, wherever mentioned, were statistically analyzed by using GraphPad Prism 8.0.2 software (GraphPad Software Inc., San Diego, CA, USA). Results are expressed as mean ± SD (*n* = 3 to 15) for independent experiments.

## 3. Results and Discussion

### 3.1. Preformulation Studies

#### 3.1.1. Powder X-ray Diffraction

**Figure 1:**
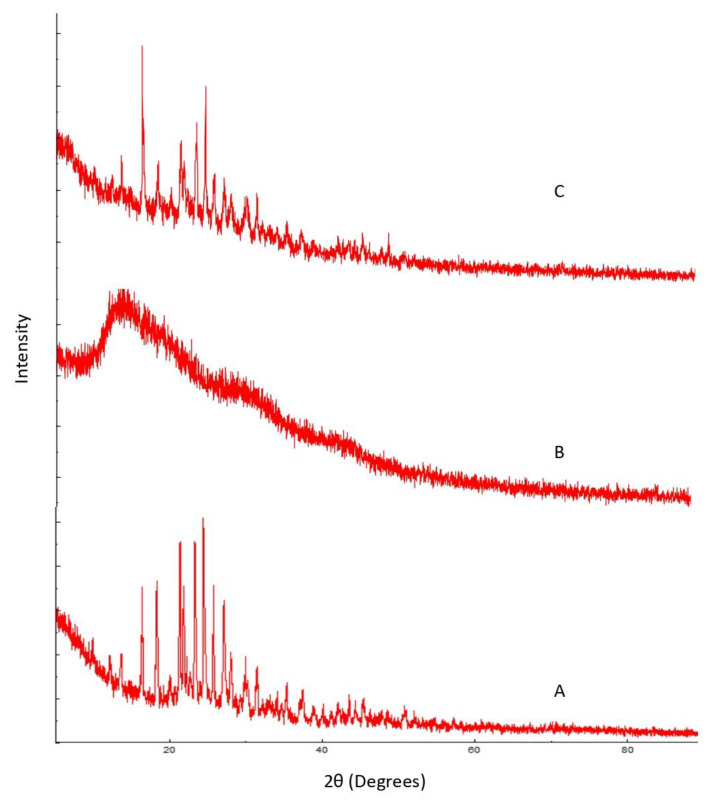
XRD overlain spectra of (**A**) pure LCZ, (**B**) Eudragit RS 100, and (**C**) physical mixture of LCZ with Eudragit RS 100.

Powder X-ray diffraction study was implied to evaluate the crystalline structure and characteristics of pure drug LCZ, polymer (Eudragit RS 100), and their physical mixture. The overlain diffractograms are shown in Figure 1. The diffractogram of pure LCZ exhibited sharp characteristic peaks of the drug with high intensity at 13.5°, 14.6°, 17.4°, 18.7°, and 21.2° (Figure 1A). This indicates that the procured and the used drug was crystalline in nature. The Eudragit RS 100 diffractogram showed less intense peaks at 12.13°, 13.45° 14.20°, 19.30°, 21.14°, and 27.1°, which is suggestive of amorphous characteristics (Figure 1B). The X-ray diffractogram recorded for the physical mixture of LCZ and Eudragit RS 100 revealed relatively less crystallinity with respect to pure LCZ and exhibited comparatively less intense characteristic drug peaks at 5.2°, 13.2°, 14.52°, and 20.28° (Figure 1C). However, the peaks of the polymer did not show any significant change, which corroborates that either the drug might have gotten entrapped in the polymer shells or, owing to the dilution effect of the polymer, the pure LCZ has gained a significantly amorphous nature [34,35,36].

#### 3.1.2. Differential Scanning Calorimetry (DSC)

**Figure 2:**
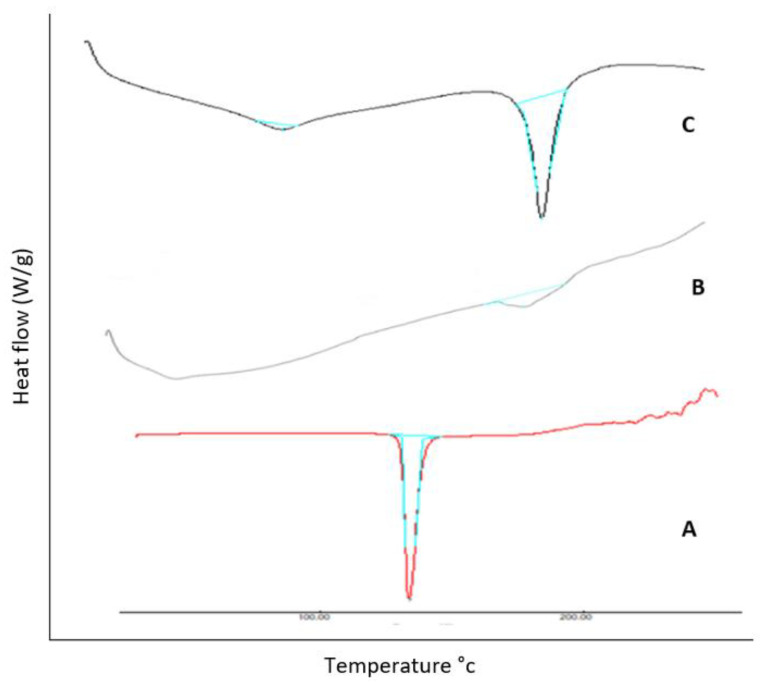
Overlain DSC thermograms of (**A**) pure LCZ (**B**) Eudragit RS 100, and (**C**) physical mixture of LCZ and Eudragit RS 100.

LCZ thermogram exhibited a wide endothermic peak at 138.07 °C (Figure 2A) corresponding to the evolution of the sample’s moisture. The distinguished melting peak at 182.91 °C manifested the natural crystalline state of the polymer (Eudragit RS 100) (Figure 2B). The characteristic endothermic peaks of both drug and polymer were observed at their authentic locations in case of the physical mixture (Figure 2C), confirming that there was almost no interaction between the drug LCZ and the polymer.

#### 3.1.3. Fourier-Transform Infrared (FTIR) Spectroscopy

**Figure 3:**
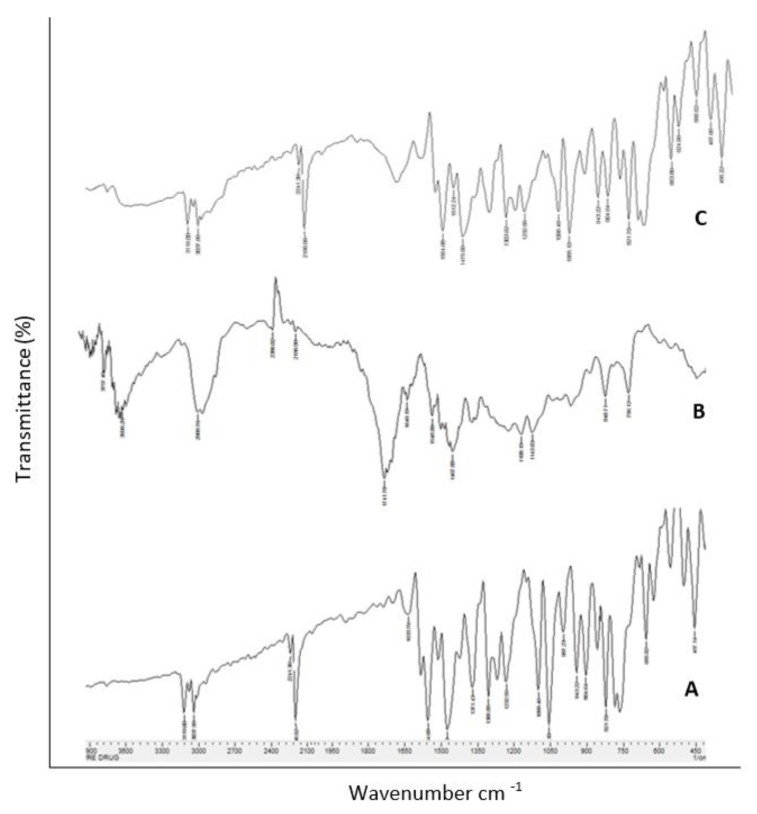
FT-IR overlain spectra of (**A**) pure LCZ, (**B**) Eudragit RS 100 and (**C**) physical mixture of LCZ and Eudragit RS 100.

FTIR spectroscopy was used to investigate the interactions between the polymer Eudragit RS 100, the drug LCZ, and their physical mixture. The LCZ OH range of FTIR generally demonstrates absorption peaks at approximately 3119 cm^−1^, 3037 cm^−1^, and 1090 cm^−1^ (Figure 3A), which could be attributed to the stretching vibrations of OH, CH, and CO, respectively. The drug powder exhibited a prominent peak at 31,190 cm^−1^, which corresponded to the stretching vibrations of OH. Bands at 3300–3000 cm^−1^, 1674–1290 cm^−1^, and 1360–1250 cm^−1^ were assigned to the alkane CH, C=C aromatic, and aryl C−N stretches (Figure 3B). For the physical mixture of LCZ and Eudragit RS100, the spectrum of the physical mixture exhibited all the characteristic peaks of the drug and the polymer (Figure 3C). There was no appearance of any new peaks or disappearance of characteristic LCZ and polymer peaks verifying the lack of any chemical interactions between the LCZ and Eudragit RS 100 [37]. From all these noted results, the high degree of compatibility between the drug and polymer used had been concluded.

### 3.2. Optimization Using Mathematical Model

**Figure 4:**
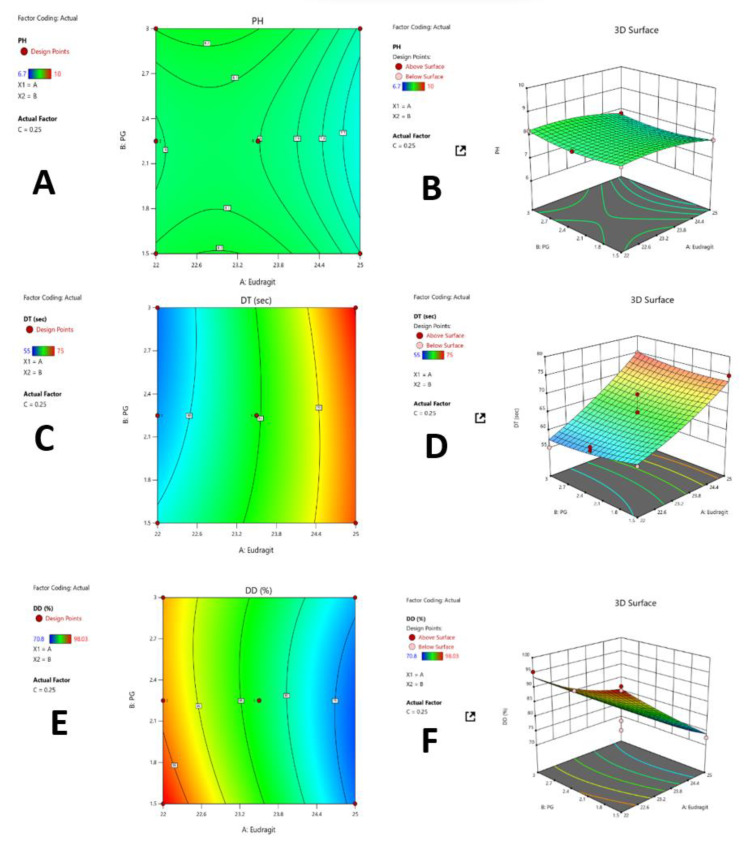
Contour and 3D response surface plot for independent variables effects on the response pH (**A**,**B**), drying time (**C**,**D**), and on drug diffusion (**E**,**F**).

Based on the factors assessed during screening trials, three parameters (drying time, pH, and drug diffusion) were selected for formulation optimization by using Design Expert software version 13. A total of 17 experimental runs were generated by using the Box–Behnken method for formulations with four factors (Table 2). Three-dimensional graphs of the response surface were plotted for drying time, pH, and drug diffusion (Figure 4). ANOVA and statistical analysis of results was performed to determine significant factors depending on the *p*-value. The factors (F1-F4) were found significant because it displayed a *p*-value less than 0.05.

#### 3.2.1. ANOVA Results

Using the response surface linear model, the f-value of 3241.58, 5.18, 4.40 for pH, drying time and drug diffusion, were found to be significant, respectively. The final regression equation determines the effect of each factor on the pH, drying time, and drug diffusion. The plus sign indicated the positive effect and the minus sign indicated the negative effect on the dependent variable. As per the obtained equations, the concentration of polymer, plasticizer, and the alkalizing agent has a positive effect on the pH and drug release. Furthermore, the response equation indicated a negative effect of the polymer, plasticizer, and alkalizing agent concentration on the drying time of the FFS.

#### 3.2.2. Identification of Optimized Formulation

The formulation was optimized based on the responses using Design Expert software (Version 13). Based on the results obtained from different responses (like drying time, pH, and drug diffusion), F-14 was selected as an optimized formulation with optimum drying time (60 s), viscosity, pH (8.1), and 98.0 ± 0.2% of drug diffusion at 420 min (Supplementary Table S2).

#### 3.2.3. Graphical Representation

The contour (Figure 4A,C,E) and 3D graphs (Figure 4B,D,F) of the response surface were plotted to represent the effects of independent variables on the dependent variables or responses and obtained graphs are presented.

### 3.3. Evaluation and Characterization of Polymeric Spray Solution

#### 3.3.1. Drying Time

The drying time of formulations was found to be in the range of 55 ± 0.04 to 75 ± 0.08 s. As the concentration of the polymer is increasing, the drying time also increases. Optimized formulation F14 displayed the desired drying time, which revealed good in situ film-forming properties for topical application. The noted drying time values for the formulations F1 to F17 are shown in Table 3.

#### 3.3.2. pH

The pH of all the formulation was in the range of 8 to 10, which are supposed to be suitable to pass up the threat of nuisance on application to the skin. As the amount of alkalizing agent was increased the pH of the solution was also increased and contributed to the adhering properties of the topical film. The noted pH values for the formulations F1 to F17 are shown in Table 3. Optimized formulation F14 exhibited the desired pH, which revealed a good in situ film-forming property for topical application and long-term retention onto the skin.

#### 3.3.3. Viscosity

Moderate viscosity is a desired and preferable flow characteristic for the in situ topical FFS. High or low viscosity results in breaking or nonuniform film formation; hence viscosity is one of the most significant rheological parameters that needs to be considered while preparing polymeric in situ films. Viscosity affects the physical characteristics, application, use, and in vivo performance of the topical FFS [22,38,39]. Viscosity is the major significant factor that determines the drying time of the film post application and greatly affects its retention time onto the skin. The viscosity of the prepared polymeric film solution was found in the range of 14 cP to 19 cP. Optimized formulation F14 was not highly viscous and flowable at room temperature, and exhibited sufficient viscosity so as to form an intact, sufficiently thick and stable in situ polymeric film post topical application. The noted viscosity values for the formulations F1 to F17 are shown in Table 3.

#### 3.3.4. Tensile Strength and Folding Endurance

Tensile strength was calculated for each film by using Equation (1), where the load at break was 1.08 kg and the strip width was 2 mm. It has been experiential that an increase in the concentration of polymer also led to an increase in the thickness and folding endurance of the film (Table 3). However, the film thickness and folding endurance also depends on the quantity of the solution sprayed or applied. Optimized formulation F14 exhibited optimal tensile strength and folding endurance, which further confirmed a good film-forming property and performance of in situ topical FFS.

#### 3.3.5. SEM Study

**Figure 5:**
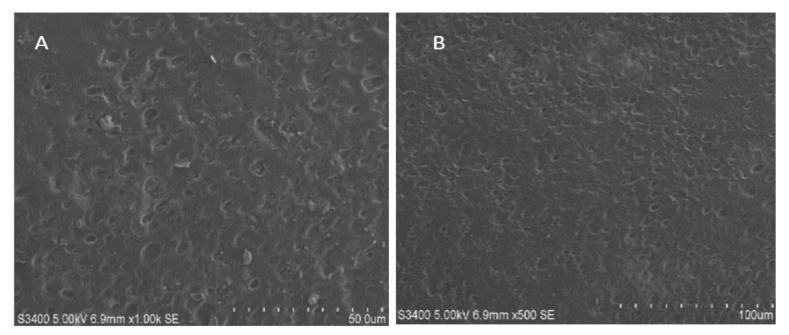
Scanning electron micrographs of optimized LCZ in situ FFS film sample at (**A**) 1000× magnification and (**B**) 5000× magnification.

Morphological investigations of LCZ in situ FFS optimized formulation (F-14, dried film) and placebo in situ FFS formulation (dried film) were carried out with the help of SEM. Captured SEM images are depicted in Figure 5A,B, which revealed that the prepared in situ FFS solution films exhibited a smooth texture and surface without any surface cracking or inconsistency. No residual, intact crystals of LCZ, particulate aggregation, and/or adsorption onto the film surfaces were observed; establishing the fact that LCZ and Eudragit RS 100 has entirely formed the in situ film matrix enclosing the uniformly distributed drug.

#### 3.3.6. Rheology Study

**Figure 6:**
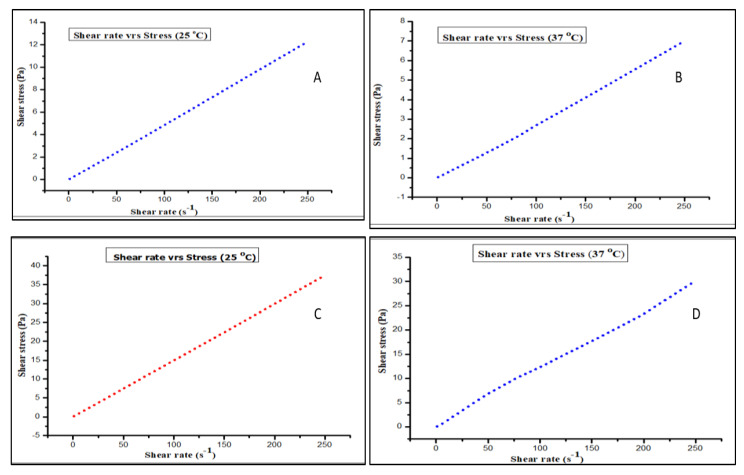
Recorded rheograms of (**A**) placebo FFS at 25 °C (dotted red line), (**B**) placebo FFS at 37 °C (dotted blue line), (**C**) optimized LCZ-based FFS at 25 °C (dotted blue line), and (**D**) optimized LCZ-based FFS at 37 °C (dotted blue line).

To evaluate the thixotropy of the solution, the flow method was implied. A solution will repeatedly pass through the sprayer nozzle if it has specific flow characteristics. When sprayed, the film-forming solution is pressed and made thinner by the flowing property before returning to its normal viscosity (stress is lost). Testing the rheological behavior of any formulation is essential for topical medication administration. For it to work, molecules must be delivered into or through the skin [40]. Moderate viscosity is the preferred film-forming solution flow characteristic, because high or low viscosity might lead to uneven film formation. The relationship between the shear stress and shear rate is explained by the Ostwald–de Waele model or power-law equation,

σ = K × γ^n^, (3)

where σ represents the shear stress (Pa), K represents the consistency index (Pa s^n^), γ represents the shear rate (s^−1^), and *n* represents the flow behaviour index. If the fluid is Newtonian in nature, *n* = 1, K becomes the fluid’s viscosity η (Pa s). To investigate the influence of polymer proportion on viscosity, rheological studies of optimized LCZ FFS and placebo FFS were performed. The recorded rheograms at 25 °C, and 37 °C have depicted a linear increase in the shear stress with an increasing shear rate amid the line passing through the origin. Thus, these outcomes indicate that both the prepared placebo formulation (Figure 6A,B) and optimized formulation (Figure 6C,D) exhibit a Newtonian type of flow. The noted rheological analysis results for placebo and optimized LCZ-based topical FFS are quoted in Table 4.

#### 3.3.7. Container Evaluation

The volume delivered per actuation and spray angle for the used spray bottle was evaluated, and the observed results are quoted in Table 5.

#### 3.3.8. In Vitro Diffusion Study

**Figure 7:**
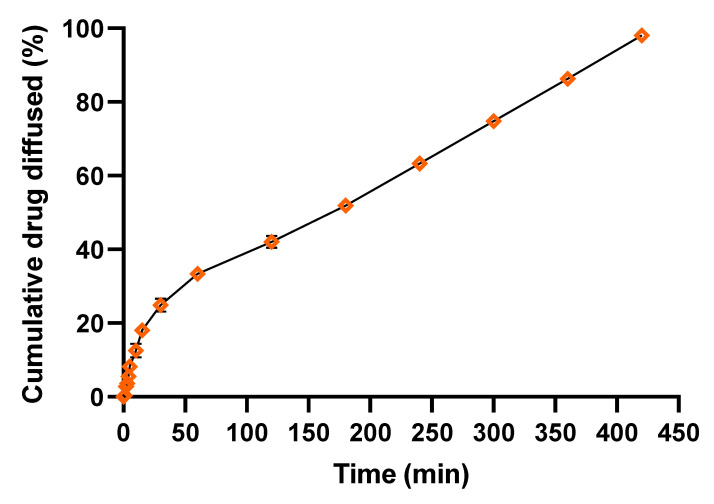
In vitro drug diffusion profile of optimized film forming solution formulation F-14.

Diffusion studies of prepared polymeric in situ FFS sprays were carried out for a period of 7 hrs. The cumulative amount of drug diffused varied based on the composition of FFS. It has been noted that almost 50% of the drug was diffused in the first 180 min itself and formulation F-14 exhibited maximum drug release up to 98.0 ± 0.2% at the end of 420 min (Supplementary Table S3). The result also indicated that there was no significant impact of polymer concentration on drug diffusion across the cellophane membrane. The percentage cumulative drug diffused at different time intervals from the optimized formulation F-14 is plotted and presented in Figure 7.

#### 3.3.9. In Vitro Antifungal Studies

The in vitro antifungal properties of polymeric solution were studied by using Sabouraud dextrose agar (SDA) medium against the *Candida albicans* strain (ATCC90028).

**Figure 8:**
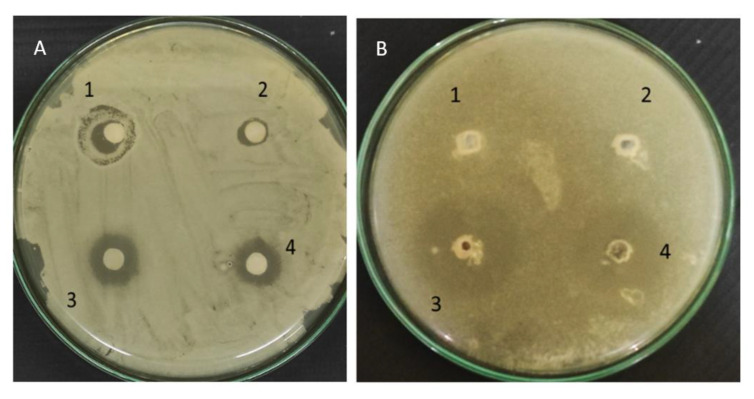
Antifungal activity of given sample against *Candida albicans* by (**A**) the disc-diffusion method and (**B**) the well-diffusion method, wherein (1) placebo, (2) placebo (alkaline), (3) formulation, and (4) drug (10 mg/mL).

The antifungal activity of the solution in terms of the zone of inhibition is presented in Table 6, and the photographic images of the zone of inhibition are shown in Figure 8. The zone of inhibition (ZOI) for the respective sample formulation was found to be greater than the positive control group (formulation) and no ZOI in the negative control (placebo). It has been experiential that the polymeric solution with the concentration of 10,000 µg/mL has effectively inhibited *C. albicans* growth. The ZOI was 9 ± 0.02 mm for the optimized formulation and 7 ± 0.02 mm for the alkaline placebo formulation. For the well-diffusion method, both drug and alkaline polymeric solutions have shown the same ZOI. The noted ZOI was 10 ± 0.04 mm and 10 ± 0.02 mm for formulation and drug, respectively, via the well-diffusion method. These results indicated the effectiveness of the sample formulation against *C. albicans* in comparison to the placebo and alkaline placebo formulations. Hence, the noted results established the accuracy of dose in the spray solution, and thus the further use of such FFS technology-based optimized preparation for the eradication of topical fungal infections.

#### 3.3.10. Stability Studies

Stability studies were conducted for a short-term period of 28 days. The optimized formulated polymeric FFS F-14 was found to be stable from the stability studies data obtained post 28 days (Supplementary Table S4). The parameters like the viscosity of the polymeric solution, the drying time of film after spraying, pH of the solution and in vitro drug-diffusion study outcomes were 14 ± 0.02 cP, 65 ± 02 s, 8 ± 0.02 and 97.18 ± 0.2%, respectively. These results showed that there was as such no impact or greater deviation on any of the performed evaluation parameters during the study period. Consequently, from the studies, it was proven that the developed and optimized polymeric FFS F-14 formulation was stable up to a period of 28 days, and further studies were to be undertaken for determining the long-term stability of the said formulation.

## 4. Summary, Conclusions and Future Directions

This study concludes that luliconazole (LCZ) containing alkaline polymeric spray solution was successfully formulated, implying a quality by design (QbD) approach. Drug (luliconazole) and polymer (Eudragit RS-100) have showed good compatibility in pre-formulation studies (FTIR, DSC, and XRD) and have depicted that there were no drug-excipient interactions. Evaluation for viscosity of the solution, drying time after application, transparency, pH, surface characterization of film, folding endurance, tensile strength and drug diffusion by using cellophane membrane were carried out by DoE Box–Behnken design. Based on the mathematical optimization approach and evaluation parameters performed, F14 was found to be an optimized formulation with optimum viscosity (14.98 cP), drying time (65 s), and drug diffusion (98 ± 0.2%). In vitro antifungal studies against *Candida albicans* showed improved ZOI (9 ± 0.2 mm) in comparison to the marketed formulation of LCZ. In vitro results revealed the activity of the above-tested formulations in the following order: optimized polymeric spray solution > alkaline blank formulation > blank formulation. At the end, the short-term stability study was conducted for 28 days, and formulation was found to be stable in all aspects for the said duration. Despite many available topical drug-delivery approaches, patient compliance and drug targeting at the desired site with optimum concentration are still concerns in developing effective treatment regimens. Thus, our current study proposes film-forming solutions as a newfangled drug-delivery approach that has great potential for surpassing the limitations of the other dosage forms like topical hydrogels and films. Moreover, it suggests that such systems provide a mean of delivering the drugs at a precise and controlled rate. Furthermore, it has been established that the drug release and drug delivery through these systems can be modulated by changing concentrations of film-forming polymers, plasticizers, additives, and even the model drugs. In a nutshell, FFSs, owing to their unique and versatile features, would be a preferable delivery system in effective eradication of an array of topical infections including topical fungal infections.

## Figures and Tables

**Table 1:** Independent and dependent variables with their levels and constraints, respectively.

Variables	Level	
Independent Variables	Low [−1]	High [+1]
Eudragit RS 100 (%)	22	25
PG (%)	1.5	3
Luliconazole (%)	1	1
Ethanol (%)	100	100
Sodium bicarbonate (%)	0.25	0.27
**Dependent Variables**	**Constraints**	
Drying time (s)	Minimize	
pH	Maximize	
Drug diffusion (%)	Maximize	

**Table 2:** Composition of in situ film-forming solution and details of optimization by DoE.

Run	Factor 1 ERS 100 (%*w*/*w*)	Factor 2 PG (%*w*/*w*)	Factor 3 Na_2_CO_3_ (%*w*/*w*)	Factor 4 LCZ (%*w*/*w*)	Factor 5 Ethanol (%*w*/*w*)	Response 1 pH	Response 2 DT (s)	Response 3 DD (%)
**1**	23.5	1.5	0.27	1	100	10 ± 0.2	65 ± 0.21	88.7 ± 0.2
**2**	23.5	3	0.27	1	100	10 ± 0.2	67 ± 0.21	85.24 ± 0.2
**3**	23.5	2.25	0.25	1	100	8 ± 0.5	65 ± 0.21	75.23 ± 0.5
**4**	23.5	2.25	0.25	1	100	8 ± 0.5	60 ± 0.08	85.4 ± 0.5
**5**	25	1.5	0.25	1	100	7.8 ± 0.2	75 ± 0.08	72.62 ± 0.2
**6**	23.5	2.25	0.25	1	100	8 ± 0.2	65 ± 0.06	90.45 ± 0.5
**7**	23.5	1.5	0.23	1	100	7 ± 0.5	65 ± 0.04	86.77 ± 0.2
**8**	23.5	2.25	0.25	1	100	8 ± 0.2	70 ± 0.06	78.56 ± 0.5
**9**	25	3	0.25	1	100	7.8 ± 0.2	75 ± 0.06	76.6 ± 0.2
**10**	23.5	3	0.23	1	100	7 ± 0.5	65 ± 0.08	82.75 ± 0.5
**11**	25	2.25	0.23	1	100	6.7 ± 0.2	70 ± 0.06	78.23 ± 0.2
**12**	22	2.25	0.25	1	100	8 ± 0.2	60 ± 0.04	93.45 ± 0.5
**13**	25	2.25	0.27	1	100	9.1 ± 0.2	75 ± 0.08	70.8 ± 0.2
**14**	22	1.5	0.25	1	100	8.1 ± 0.2	60 ± 0.04	98.0 ± 0.2
**15**	23.5	2.25	0.25	1	100	8 ± 0.2	62	85.91 ± 0.5
**16**	22	2.25	0.25	1	100	8 ± 0.2	59 ± 0.08	93.45 ± 0.5
**17**	22	3	0.25	1	100	8.2 ± 0.2	55 ± 0.04	95.28 ± 0.2

**Table 3:** Evaluation of formed polymeric spray solution for different standard parameters.

Run	pH	Viscosity (cP)	Drying Time (s)	Upper Surface	Transparency	Thickness (mm)	Folding Endurance	Tensile Strength (kg/cm^2^)
F-1	10 ± 0.2	14.98 ± 0.12	65 ± 0.21	Smooth	++	0.16 ± 0.02	53 ± 2	3.16 ± 0.03
F-2	10 ± 0.2	15.01 ± 0.21	67 ± 0.21	Smooth	++	0.16 ± 0.01	57 ± 2	3.16 ± 0.03
F-3	8 ± 0.5	14.97 ± 0.28	65 ± 0.21	Smooth	++	0.15 ± 0.02	50 ± 2	3.36 ± 0.03
F-4	8 ± 0.5	14.98 ± 0.31	60 ± 0.08	Smooth/white residue after drying	++	0.15 ± 0.01	45 ± 2	3.36 ± 0.03
F-5	7.8 ± 0.2	15.20 ± 0.12	75 ± 0.08	Smooth	++	0.18 ± 0.02	50 ± 2	2.83 ± 0.03
F-6	8 ± 0.2	15.34 ± 0.21	65 ± 0.06	Smooth	++	0.16 ± 0.01	53 ± 2	3.16 ± 0.03
F-7	7 ± 0.5	15.15 ± 0.28	65 ± 0.04	Smooth	++	0.16 ± 0.02	58 ± 2	3.16 ± 0.03
F-8	8 ± 0.2	15.37 ± 0.31	70 ± 0.06	Smooth/white residue after drying	++	0.17 ± 0.01	51 ± 2	3.15 ± 0.03
F-9	7.8 ± 0.2	18.68 ± 0.21	75 ± 0.06	Smooth	++	0.20 ± 0.02	48 ± 2	2.56 ± 0.03
F-10	7 ± 0.5	19.24 ± 0.28	65 ± 0.08	Smooth	++	0.19 ± 0.01	50 ± 2	2.67 ± 0.03
F-11	6.7 ± 0.2	19.25 ± 0.21	70 ± 0.06	Smooth	++	0.19 ± 0.01	52 ± 2	2.76 ± 0.03
F-12	8 ± 0.2	19.20 ± 0.21	60 ± 0.04	Smooth/white residue after drying	++	0.20 ± 0.02	54 ± 2	2.56 ± 0.03
F-13	9.1 ± 0.2	14.99 ± 0.21	75 ± 0.08	Smooth	++	0.16 ± 0.01	53 ± 2	3.16 ± 0.03
F-14	8.1 ± 0.2	14.98 ± 0.21	60 ± 0.04	Smooth	++	0.16 ± 0.01	57 ± 2	3.36 ± 0.03
F-15	8 ± 0.2	14.99 ± 0.28	62 ± 0.06	Smooth	++	0.16 ± 0.01	58 ± 2	3.16 ± 0.03
F-16	8 ± 0.2	14.97 ± 0.28	59 ± 0.08	Smooth	++	0.15 ± 0.02	53 ± 2	3.36 ± 0.03
F-17	8.2 ± 0.2	14.98 ± 0.28	55 ± 0.04	Smooth	++	0.15 ± 0.01	55 ± 2	3.36 ± 0.03

* Mean ± SD, (*n* = 3); ++, completely transparent; +, acceptable; −, blurred.

**Table 4:** Rheological analysis of placebo and optimized LCZ based topical FFS.

Temperature	Flow Type	η (Pa s)/k (Pa s^n^) ^#^
Placebo in situ film		
25 °C	Newtonian	0.5775
37 °C	Newtonian	0.5508
Optimized Luliconazole based topical in situ film		
25 °C	Newtonian	0.52779
37 °C	Newtonian	0.51572

^#^ For the Newtonian flow-type ‘*n*’ is always 1.

**Table 5:** Results noted for container evaluations.

Parameter 1		Parameter 2	
SI. No.	Volume of Solution upon Each Actuation (mL) *	SI. No.	Spray Angle *
1	0.25 ± 0.05 mL	1	80 ± 2

* Mean ± SD (*n* = 3).

**Table 6:** Zone of inhibition noted against *Candida albicans*.

Sample	Zone of Inhibition (mm)	
	Disc Method	Well Diffusion
Placebo	7 ± 0.02	-
Placebo (alkaline)	5 ± 0.03	-
Formulation	9 ± 0.02	10 ± 0.04
Drug	8 ± 0.04	10 ± 0.02

## Data Availability

Data will be made available on request.

## Supplementary Materials

The following supporting information can be downloaded at: https://www.mdpi.com/article/10.3390/polym15041003/s1, Supplementary Figure S1: UV spectrum and lambda max of LCZ pure drug; Supplementary Figure S2: Calibration curve of LCZ pure drug *via* UV spectrophotometry; Supplementary Table S1: Concentration and absorbances of LCZ pure drug *via* UV spectrophotometry, Supplementary Table S2: Final composition and/or formula for the optimized formulation F-14, Supplementary Table S3: In vitro drug diffusion study results of optimized film forming solution F-14, Supplementary Table S4: Stability studies data of the optimized polymeric FFS F-14.
