# Supplementary material for: Newfangled Topical Film-Forming Solution for Facilitated Antifungal Therapy: Design, Development, Characterization, and In Vitro Evaluation
Source: Polymers (Basel). 2023 Feb 17;15(4):1003. doi: 10.3390/polym15041003 (PMC9961451; doi:10.3390/polym15041003)
Supplement: Supplementary file 1 [file polymers-15-01003-s001.zip › polymers-2135270-supplementary.pdf]

polymers

MDPI

Supplementary Data

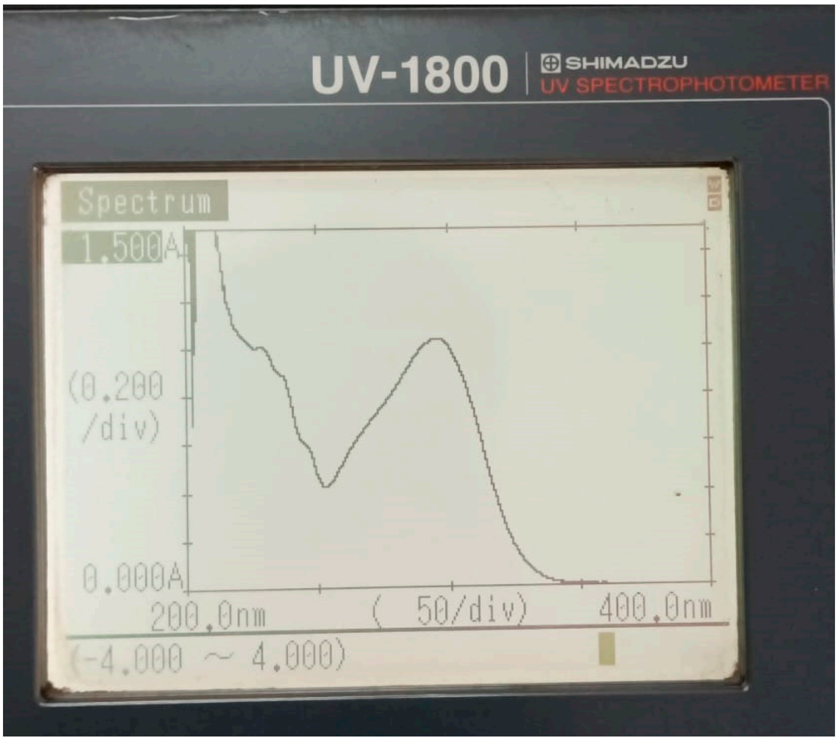

Supplementary Figure S1: UV spectrum and lambda max of LCZ pure drug.

Supplementary Table S1: Concentration and absorbances of LCZ pure drug *via* UV spectrophotometry.

Concentration of LCZ (µg/mL)	Absorbance at 290 nm
0	0
5	0.112±0.01
10	0.223±0.04
15	0.357±0.031
20	0.481±0.02
25	0.571±0.05
30	0.693±0.08

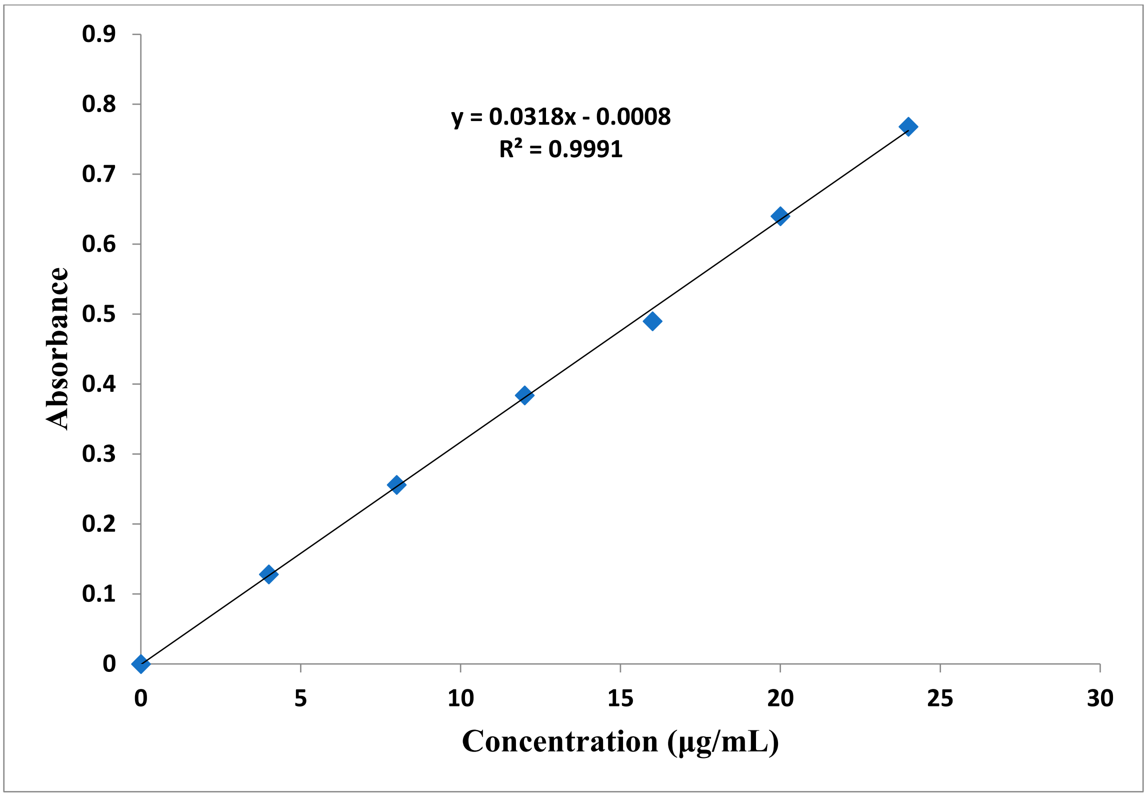

Supplementary Figure S2: Calibration curve of LCZ pure drug *via* UV spectrophotometry.

Supplementary Table S2: Final composition and/or formula for the optimized formulation F-14.

Eudragit RS-100 (%)	Propylene Glycol (%)	Sodium bi-carbonate (%)	Ethanol (ml)	Luliconazole (%)
22%	1.5%	0.25%	10	1%

Supplementary Table S3: In vitro drug diffusion study results of optimized film forming solution F-14.

Time (min)		0	1	2	3	4	5	10	15	30	60	120	180	240	300	360	420
Cumulative drug diffused (%)	F-14	0	0.34	2.77	3.66	5.58	8.24	12.58	18.04	24.9	33.36	42.07	51.9	63.33	74.83	86.3	98.0

Supplementary Table S4: Stability studies data of the optimized polymeric FFS F-14.

Time (Days)	Viscosity* (cP)	Drying time* (sec)	pH*	Cumulative drug diffused* (%)
0	14 ±0.01	60±02	8±0.03	98.48±0.2
7	14 ±0.02	60±02	8±0.02	98.07±0.2
14	13±0.02	65±04	8±0.03	97.79±0.6
21	14±0.01	60±02	8±0.01	97.55±0.3
28	13±0.02	63±01	8±0.02	97.18±0.2

*Mean ± SD, (*n* = 3)
